# Bottom-up, integrated -omics analysis identifies broadly dosage-sensitive genes in breast cancer samples from TCGA

**DOI:** 10.1371/journal.pone.0210910

**Published:** 2019-01-17

**Authors:** Bobak D. Kechavarzi, Huanmei Wu, Thompson N. Doman

**Affiliations:** 1 Indiana University Purdue University Indianapolis, School of Informatics and Computing, Indianapolis, IN, United States of America; 2 Eli Lilly, Indianapolis, IN, United States of America; University of South Alabama Mitchell Cancer Institute, UNITED STATES

## Abstract

The massive genomic data from The Cancer Genome Atlas (TCGA), including proteomics data from Clinical Proteomic Tumor Analysis Consortium (CPTAC), provides a unique opportunity to study cancer systematically. While most observations are made from a single type of genomics data, we apply big data analytics and systems biology approaches by simultaneously analyzing DNA amplification, mRNA and protein abundance. Using multiple genomic profiles, we have discovered widespread dosage compensation for the extensive aneuploidy observed in TCGA breast cancer samples. We do identify 11 genes that show strong correlation across all features (DNA/mRNA/protein) analogous to that of the well-known oncogene HER2 (ERBB2). These genes are generally less well-characterized regarding their role in cancer and we advocate their further study. We also discover that shRNA knockdown of these genes has an impact on cancer cell growth, suggesting a vulnerability that could be used for cancer therapy. Our study shows the advantages of systematic big data methodologies and also provides future research directions.

## Introduction

The scientific literature is replete with papers highlighting the complex interplay between chromosomal instability, aneuploidy, and cancer (e.g. [1] [2] [3] [4]). Aneuploidy, the state of having other than the canonical or “euploid” number of chromosomes—for humans, 46—is with only rare exceptions (Downs syndrome, Trisomy 18) lethal in human embryonic development [5]. By contrast, aneuploidy is observed with very high frequency in cancer, leading the eminent German biologist Theodor Boveri to speculate as early as 1902 [6] that aneuploidy might have a causative role in the disease.

Despite previous investigations, there are still important questions. Is aneuploidy a cause or a side-effect of cancer? If the former, what factors associated with aneuploidy contribute to cancer cell fitness? Are there deleterious impacts of aneuploidy in cancer and how are they mitigated during tumorigenesis? More generally, what is the broader impact of aneuploidy on gene expression and resulting phenotypes?

DNA studies have found that amplification of genomic arms such as 20q and 8q [7] [8] occur with high prevalence and have been correlative with cancer severity. Understanding how these amplifications impact changes in gene expression and protein production is of great interest. The conventional wisdom regarding gene transcription and translation has been that “dosage” *generally* correlates with product: DNA to RNA to protein. Indeed, a recent report finds no evidence for widespread dosage compensation in yeast [9].

It is customary to use mRNA transcript abundance to identify disease-associated genes, but the impact of mRNA abundance on protein production is poorly understood. Correlational methods yield weak associations, even when considering protein half-lives and other chemical properties [10–13]. Other efforts have been made to integrate mRNA dynamics (half-life and fold energy) and RNA Binding Protein (RBP) interactions with expression data in *S*. *cerevisiae* and *S*. *pombe* to aid in predicting protein production from gene expression. [14] Illustrates how sequence elements (sequence lengths, secondary structures, etc.) were used to identify protein abundance variations. Understanding how DNA, RNA, and protein interact is a non-trivial task, but considering any of these features in isolation may yield sub-optimal results. This understanding could provide crucial details about tumorigenesis, cancer evolution, and may hold clues to potential cancer treatments.

In 2015 approximately 40,000 women died of breast cancer in the US alone [15]. In an effort to better profile and understand cancer, large public efforts have been initiated to gather patient data and comprehensively investigate it. The Cancer Genome Atlas (TCGA) collects data for patients across 34 types of cancer profiled using a wide array of ‘-omics’ platforms [16]. The unprecedented availability of cancer data, like TCGA, affords insights into the genomic foundation of these lethal diseases.

In this paper, we apply big data methods in a systematic fashion to observe the impact of DNA dosage on mRNA transcript levels and subsequent protein concentrations. We identify the prevalence of dosage compensation in TCGA breast cancer samples (BRCA), highlight dosage-sensitive genes, and investigate the role of these genes in cancer cell line survival.

## Material and methods

**Fig 1:**
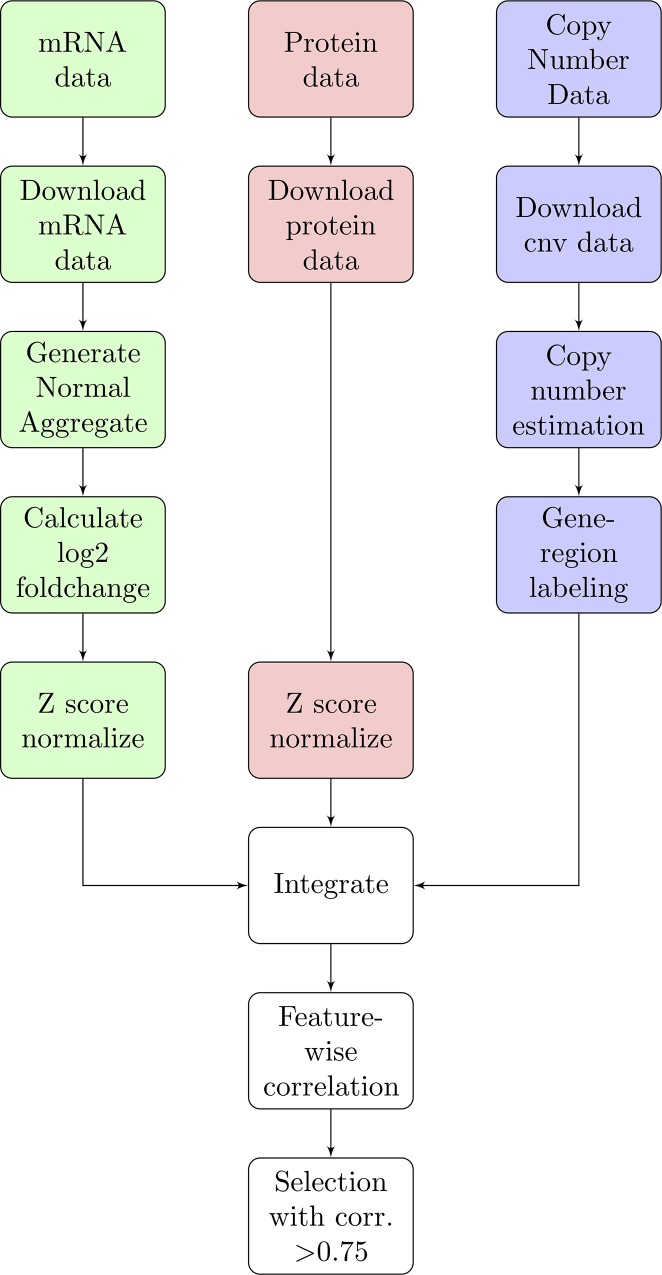
Bottom-up, integrated analysis workflow. Visual representation of the analytical workflow for identifying broadly dosage-sensitive genes. Green portions represent mRNA-based steps, red protein, blue CNV. Integrated and filtering steps are white. Briefly, data were acquired from their sources, joined with metadata, normalized, integrated, then filtered.

The data used in this study has been downloaded from multiple resources, including TCGA [17], Clinical Proteomic Tumor Analysis Consortium (CPTAC) [18], the Catalogue of Somatic Mutations in Cancer (COSMIC) [19], and Achilles short hairpin RNA or small hairpin RNA (shRNA) [20]. The data and processing approaches are briefly described below. Fig 1 illustrates the overall workflow.

### The Cancer Genome Atlas (TCGA)

RNAseq V2 data of 114 normal control patients and 1102 patients with breast invasive carcinoma (BRCA) were downloaded from TCGA. For each of the 20532 genes of each patient, the median of 114 normal values was used as an estimated baseline, which is noted as the *Normal median RSEM*. The fold change of each gene, Δ_*exp*_(*gene*), was calculated for every patient as:

$$\Delta_{exp}(gene)=log_{2}\frac{Cancer\;RSEM\;(gene)}{Normal\;median\;RSEM(gene)}$$ (Eq 1)

The corresponding patient metadata was downloaded and mapped based on sample IDs extracted from the TCGA barcode. Level 3 TCGA copy number variant (CNV) data was extracted for all available patients. The TCGA CNV pipeline transforms a CNV value into a segment mean, where *Segment mean* = *log*_2_(*CNV*/2). A copy number can be derived from the segment means by calculating 2 * (2^*segment mean*^). With this, the diploid regions will have a segment mean value of zero, amplified regions will have positive values, and deletions will have negative values.

### Clinical proteomic tumor analysis consortium (CPTAC)

Mass spectrometry (MS) data for breast invasive carcinoma was downloaded from CPTAC; these abundances were reported as the log_2_-ratio of the expression of the sample to a common, healthy pool [21]. Patient mRNA, protein, and CNV data was matched using TCGA barcode. Gene identifications for MS data were made using previously established methods [22] and were provided by CPTAC. mRNA, protein, and CNV data for a given gene were joined by gene symbol. The unshared relative protein abundance was matched to 106 patients with mRNA and protein abundance data [18].

### Gene amplification and deletion

Segment regions were mapped to the UCSC genome coordinates for the hg38 build of the human genome. For regions that covered multiple genes, the segment means counted for each gene. For genes with multiple calls, the maximum value was kept.

For genes across all 106 samples with protein and mRNA data, we observe 1,052,345 segment means in total. The average segment mean was 0.12 ± 0.02. We define a gene as amplified if its segment mean is greater than 0.2 and deleted if it is less than -0.2 [23]. Doing so we find 837,531 normal segments in the patients, 213,361 segments are amplified, and 1,453 have deletion events. Of those, 9835 genes were uniquely identified as normal, 9831 as amplified, and 1247 as deleted. Interestingly, only 15 patients of the 106 had no deletions.

To normalize protein and mRNA expression to a similar scale, a z-score normalization for protein and mRNA fold changes was performed as follows:

$$z=\frac{x_{\Delta_{exp}}-\mu_{\Delta_{exp}}}{\delta_{\Delta_{exp}}}$$ (Eq 2)

Where $x_{\Delta_{exp}}$ represents the expression of a given gene, $\mu_{\Delta_{exp}}$ the mean expression for the data set, and $\delta_{\Delta_{exp}}$ the standard deviation.

### Cancer gene profiling

The Cancer Gene Census was downloaded from COSMIC [19]. Genes labelled as amplified were selected and mapped to the results of the integrated genomics analysis to annotate correlational signatures. The Pearson correlation coefficient was calculated between protein abundance fold-change, mRNA fold-change, and CNV amplification. Any gene with all correlational scores above 0.70 is called a “Broadly Dosage-Sensitive Gene,” or **BDSG**. The stringent cutoff was selected to emphasize genes as very unique. Generally, there was poor correlational concordance among the features (Fig A in S1 File and Table A in S1 File). This is not to say the genes below this threshold may not be informative, but they are not exemplars of this particular genomic conservation.

### Achilles shRNA

Achilles shRNA knockdown data was downloaded and subset for the BDSG genes, a selection of housekeeping genes [24] and genes acting as oncosuppressors or oncogenes (S1 Table). shRNA hairpins for each gene were selected based on second-lowest log-ratio to avoid false positives. Hierarchical clustering was performed via Python clustermap function on the genes, as well as the cell types. Additional hierarchical clustering was performed excluding any cell types not related to breast models.

## Results and discussion

### Genomics and protein analysis

RSEM distributions for the 106 breast cancer patients for 20531 genes were plotted for both the cancer and healthy samples. Initial observations of RSEM values show similar quantiles suggesting that global expression distributions are similar between tumor-matched normal and tumor samples (Fig B in S1 File). This alleviates concern for the impact of any batch-effect or other temporal anomalies in sample processing.

Wilcoxon signed-rank test of gene expression between healthy and cancer samples detected that over 10,000 genes had significantly different expression (p < 0.00005). This emphasizes that the cancer transcriptome varies dramatically from normal tissue.

The log_2_ fold-change of mRNA from cancer to healthy was calculated as (Eq 1), described with a mean mRNA fold change of -9.0*10^−2^ ± 1.5 (max = 17, min = – 16). The indicates a class of genes that have minimal change and another subset that shows large changes in expression as indicated by the distribution (Fig C in S1 File).

**Fig 2:**
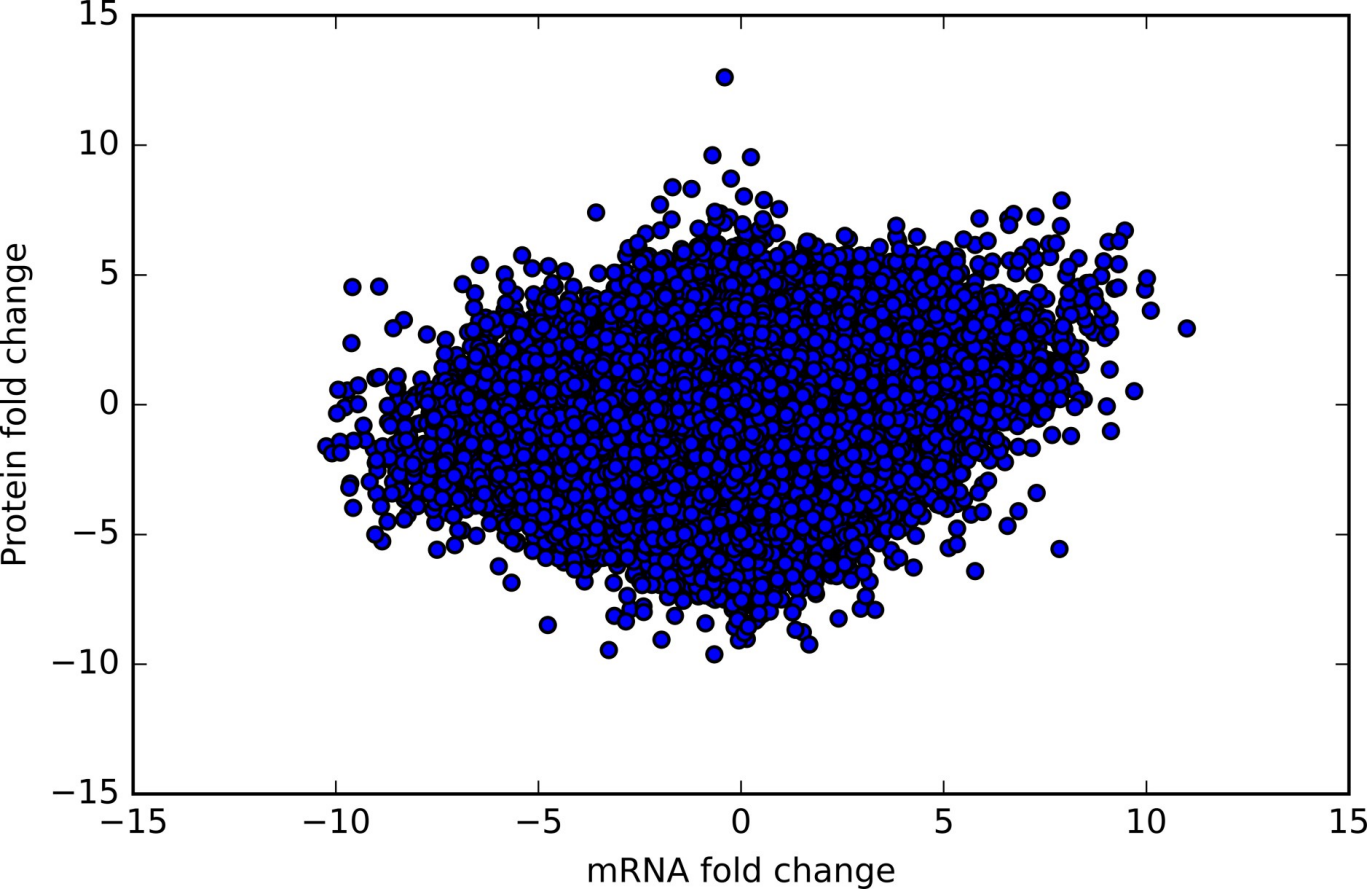
mRNA fold change versus protein fold change. This represents the matched protein (y-axis) and mRNA (x-axis) z-score normalized, log_2_ fold changes of disease to healthy samples for 106 patients and 20531 genes. Poor correlation amongst the points suggests that mRNA changes do not necessarily predict protein production changes.

Fold changes were plotted for protein expression. For proteins, the mean fold change was 3*10^−2^ ± 0.67 (max = 8.5, min = – 6.5). Since the dynamic range of these effects are not equivalent in both datasets, they were z-score normalized (Fig D in S1 File). After normalization (Fig E in S1 File), the overall distributions of mRNA and protein fold changes were not statistically different (p = 0.96, Wilcoxon Test). Therefore, our normalization practice is sufficient to integrate the datasets and attempt to find relationships. These relationships are visualized in Fig 2. The strikingly poor correlation between the two features emphasizes the difficulty in accurately inferring protein levels from given mRNA expression values. A D’Agostino’s K-squared test of the mRNA and protein correlations led to the rejection of the null hypothesis that the data was Guassian (p < 0.0005). Further examination of QQ plots conveys a trend for a mariginally lighter left tail and a heavier right tail to the distribution (Fig F in S1 File).

### mRNA and protein dysregulation relative to copy number variation

There is a strong positive trend for correlation of mRNA fold change with segment means. This is intuitive, given if there is an amplification or deletion of a genomic region transcriptional activity is impacted.

A surprising result is the volume of anti-correlated genes when comparing mRNA versus protein fold-changes (Fig G in S1 File). There are 185 genes out of the 9835 with weak, negative correlations between mRNA and protein fold-changes. A negative correlation suggests that a regulatory mechanism is either additionally suppressing or enhancing transcript abundance. We observe a number of genes which have negative correlation. These are of relatively low magnitude and lack statistical significance, but there may be underlying biological mechanisms at play that may be of interest in future studies. This notion of regulatory interactions is emphasized again by observing the correlation of protein changes to segment means (Fig H in S1 File). In this case there are generally weak correlations, supporting the concept that precursor genomic features are often not a reliable predictor of protein concentration [25].

Selecting genes and subsequent data based on the 20q chromosomal arm–a locus known to be frequently-amplified in breast cancer [26] exemplifies how DNA amplification, mRNA and protein abundance may be discordant. In the case of the gene SLPI for a sample (TCGA barcode: D8-A13Y), we see that there is strong DNA amplification, however mRNA and protein abundance is very low. SLPI encodes an antibody-producing transcript which antagonizes paclitaxel in ovarian cancer cells [27]. Conversely, we can see cases where there is concordance between amplification, mRNA abundance, and protein abundance. In the case of RIMS4, we see very high fold changes and amplification in certain members of the cohort. KM-survivability analysis [28] based on microarray data of over 1000 breast cancer patients indicates that high RIMS4 expression has a positive prognostic impact (P <4.0x10^-7^).

### Cancer gene profiling identifies broadly dosage-sensitive genes (BDSGs)

**Fig 3:**
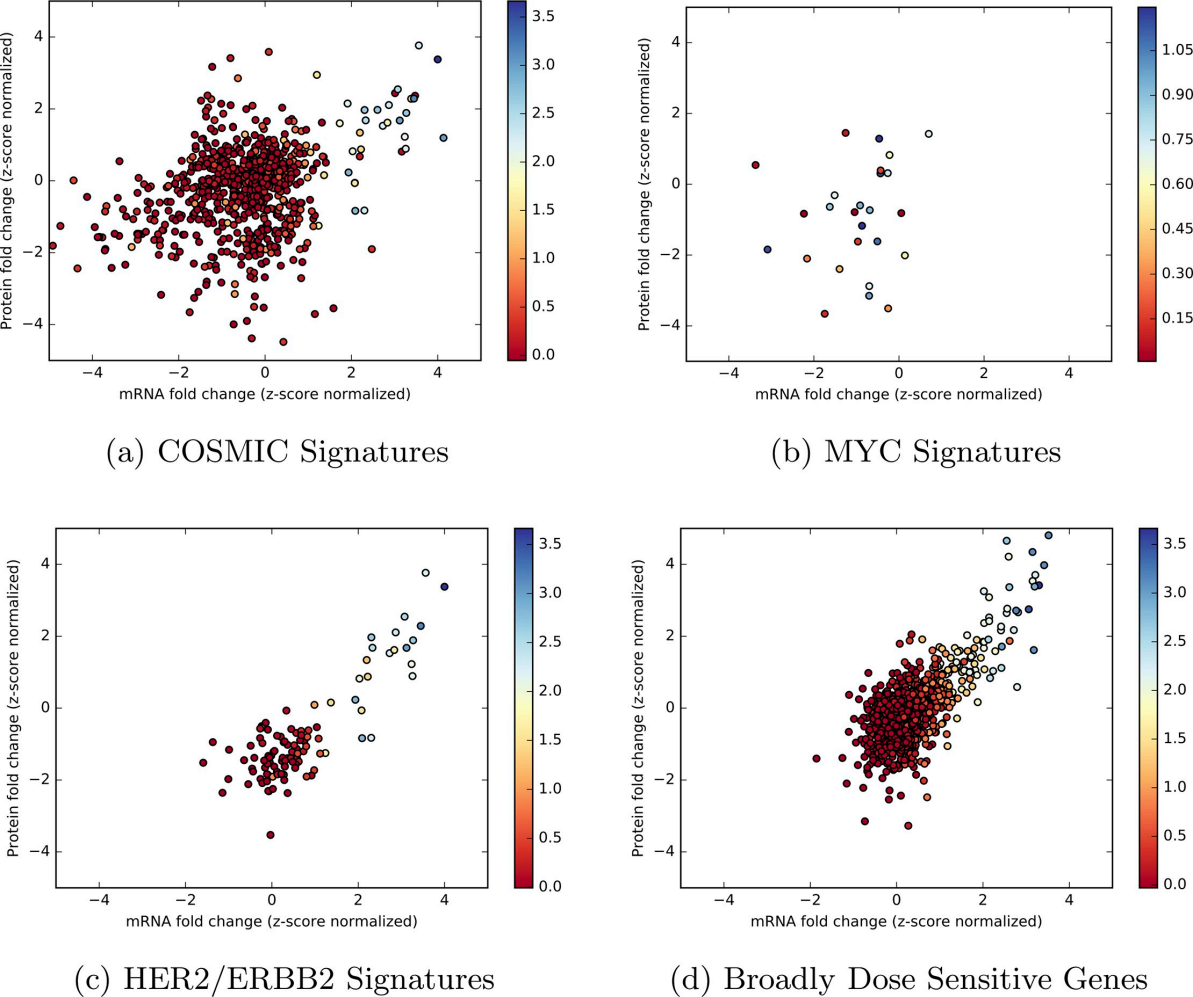
Protein vs mRNA fold changes with CNV amplification. Figures represent the z-score normalized, log2 fold change of mRNA (x-axis) versus protein (y-axis) and are colored by CNV segment mean values for samples from patients in the TCGA Breast Cancer dataset selected for: (a) Genes in the COSMIC database labelled as amplified, (b) known oncogene MYC, (c) known oncogene and BDSG HER2/ERBB2, and (d) all BDSGs. The trend in Fig 3A exemplifies how oncogenecity does not always correlate with dosage-sensitivity.

The mRNA, protein, and CNV data for genes labeled as amplified in breast cancer from COSMIC is in Fig 3A. Genes from across the genome that meet the Pearson correlation criteria (i.e., all correlations above 0.75) are displayed in Fig 3D and listed in Table 1. Among the genes in Table 1, ERBB2 (HER2, Fig 3C) is a member of this group and is a well-known oncogene. In both Fig 3C and Fig 3D, we observe a strong dosage sensitivity that is atypical across the genome. The remaining 11 genes in Fig 3D are not identified in COSMIC at all. GRB7 is a growth factor receptor that overlaps with HER2 pathways, and coexpresses with it in esophageal cancer [29]. RPS6KB1 is a kinase whose alterations have been associated with an increased risk of colorectal cancer [30]. These broadly dosage-sensitive genes (BDSGs) are observations on a seemingly rare conservation of the central dogma, yet they have minimal functional annotations in the scientific literature.

**Table 1:** Broadly dosage-sensitive genes (BDSGs).

Symbol	Location
ERBB2	17q11.2-q12
GBAS	7p12
GRB7	17q12
HEATR6	17q23.2
LANCL2	7q31.1-q31.33
PDSS2	6q21
PPFIA1	11q13.3
PPME1	11q13.4
RPS6KB1	17q23.1
SUMF2	7q11.1
TACO1	17q23.3
UBE2Z	17q21.32

### shRNA data defines the role of BDSGs in cancer cell line growth

**Fig 4:**
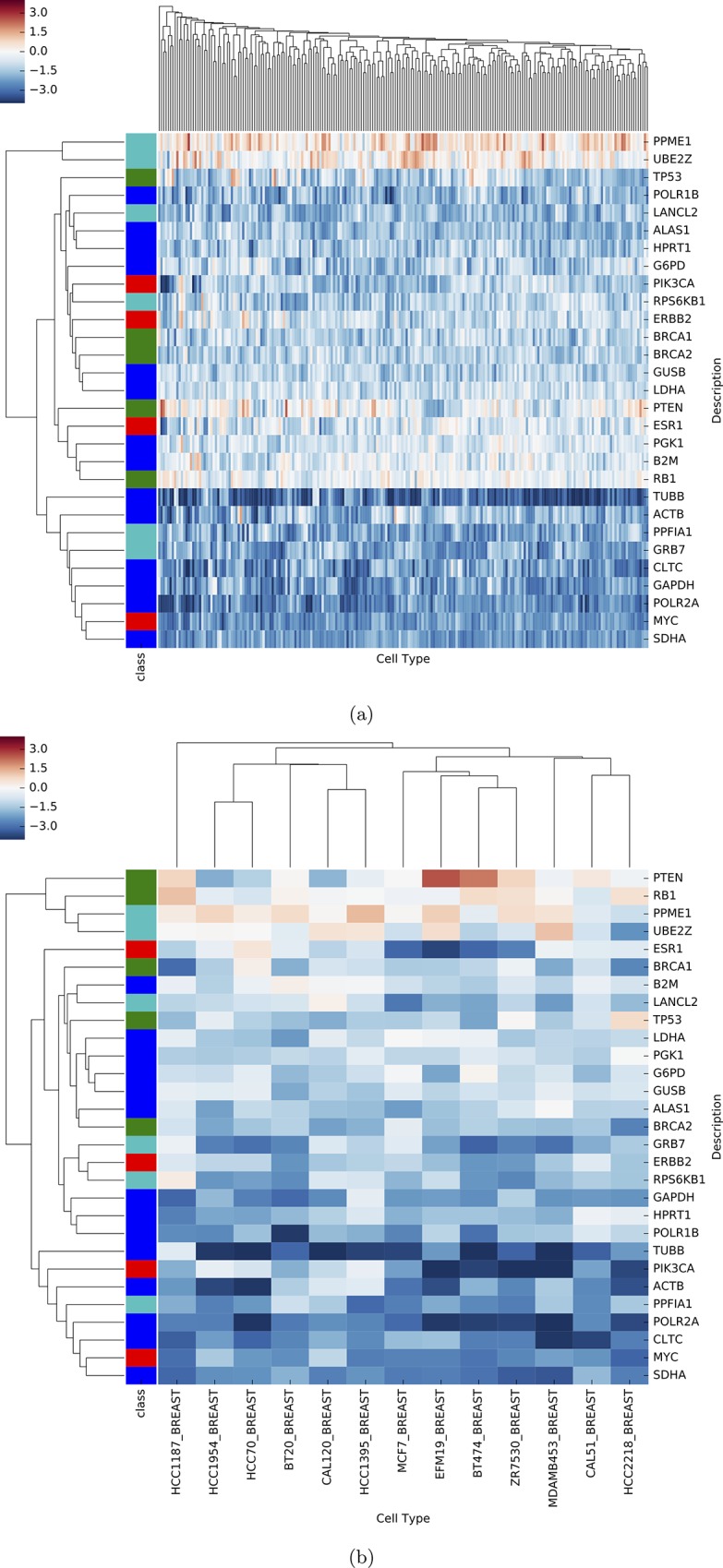
Heatmap and hierarchical clustering of shRNA knockdown. Section (a) represents all cell lines available in the Achilles project and (b) breast-specific cell lines. Rows are the selected genes: blue are housekeeping genes, red are oncogenes, green oncosupressors, and light blue are BDSGs; columns are the cell lines. Red cell values represent cellular proliferation, blue cellular death, and white no change. The clustering of ERBB2 and two BDSGs (GRB7, RPS6KB1) in (b) suggests an oncogenic role in breast cancer. PPME1 and UBE2Z signatures in (a) and (b) suggest an overall oncosuppressive role.

Fig 4 shows results of testing the impact of BDSGs on cancer cell line growth using shRNA. TUBB is a common housekeeping gene and the signature illustrated in 4a is typical of this role. When knocked down by shRNA there is a very deleterious effect on cancer cell line growth and it stands out as a singleton in both heatmaps. In Fig 4A we observe that PPME1 and UBE2Z consistently behave as tumor suppressor genes (TSGs) across all cell types; their silencing promotes cell viability. In breast cancer cell lines these genes cluster closely with PTEN and RB1 which were included as typical breast cancer TSGs. In contrast, GRB7 and RPS6KB1 have a generally negative impact on cell line viability in Fig 4A. However, when considering just breast cancer-specific cell lines, we observe that these genes cluster closely and exclusively with ERBB2. The differences in clustering behavior suggest that, unlike PPME1 and UBE2Z, GRB7 and RPS6KB1 act as oncogenes very similar to ERBB2. In fact, GRB7 is co-located with ERBB2 and may be upregulated as an adaptation to HER2 [29]. According to the breast-specific cell type clustering of shRNA data, BDSGs do not display a subtype-specific role. They are generally tumor suppressors or oncogenes across all breast cell lines.

## Conclusions

In this paper we have shown that in the TCGA breast cancer cohort there is widespread dosage compensation for the extensive aneuploidy that is observed. The dosage of DNA does not generally correlate well with mRNA, nor does the latter correlate well with protein levels. A total of 11 genes show strong correlation across all features (DNA/mRNA/protein); analogous to that of a well-known oncogene HER2 (ERBB2). We refer to these genes as “Broadly Dosage-Sensitive Genes” or BDSGs. We note they are much less characterized in the literature as to their role, if any, in cancer. We advocate further study of BDSGs to better understand their potential effects on cancer. This may lead to new therapies for cancer or biomarkers for improved cancer detection.

From shRNA data, we show that knockdown of these genes has an impact on cancer cell growth. We speculate that tumor cells adapt unusual ploidies to take advantage of amplifications and deletions that functionally implicate only subsets of genes. These tumor cells may compensate for the dosage of a large number of “passenger” genes. This may be a vulnerability that could be used for cancer therapy, for example by de-repressing mRNA and/or protein production from these passenger genes. This may leave the tumor cell with potentially catastrophic levels of unneeded molecules or disrupted biological pathways.

We also caution that there may be significant pitfalls in drawing conclusions from a single type of genomics data. For example, gene expression (mRNA) data is widely-used to infer biological pathway activation, but Fig 2 suggests this would be extremely misleading for exploring protein levels of Cancer Gene Census genes in TCGA Breast samples.

## Supporting information

S1 Table House keeping, oncosuppressor, and oncogenes selected for Achilles shRNA analysis. (XLSX)

S1 File Additional figures profiling data characteristics. (PDF)
