# Supplementary material for: Bottom-up, integrated -omics analysis identifies broadly dosage-sensitive genes in breast cancer samples from TCGA
Source: PLoS One. 2019 Jan 17;14(1):e0210910. doi: 10.1371/journal.pone.0210910 (PMC6336338; doi:10.1371/journal.pone.0210910)
Supplement: S1 File — (PDF) [file pone.0210910.s002.pdf]

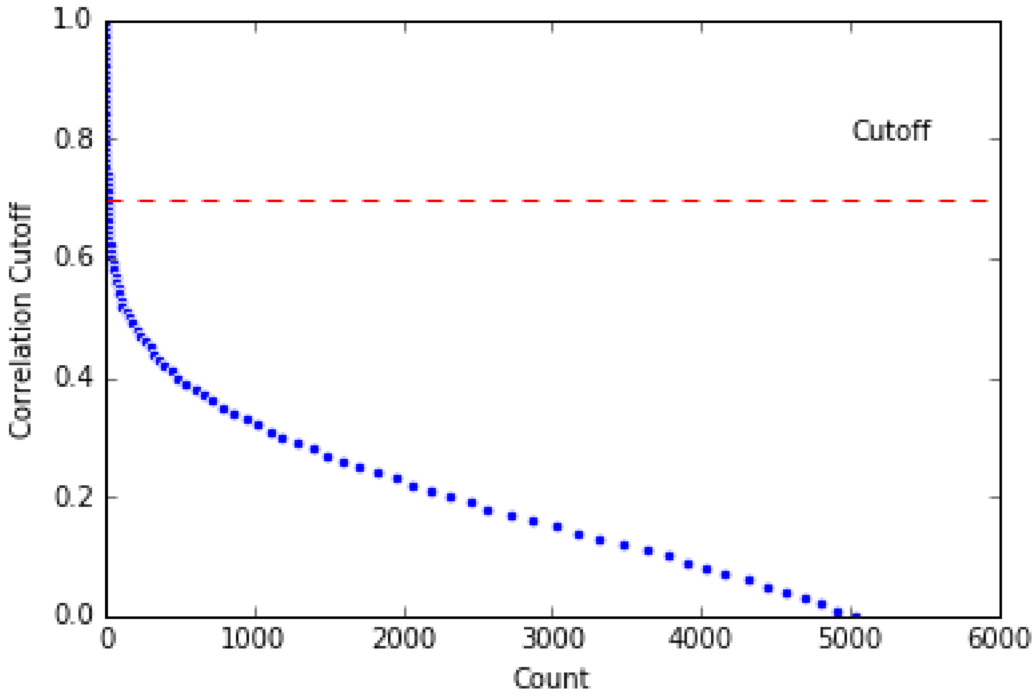

Fig A. Correlation coefficient cutoff

Scatter plot of correlation threshold cut offs and subsequent member counts. The red, dotted line indicates cutoff used for BDSG identification for the study.

Correlation Cutoff	Count
0.99	0
0.98	0
0.97	0
0.96	0
0.95	0
0.94	0
0.93	0
0.92	0
0.91	0
0.90	0
0.89	0
0.88	0
0.87	0
0.86	0
0.85	0
0.84	1
0.83	1
0.82	2

0.81	2
0.80	2
0.79	3
0.78	4
0.77	4
0.76	5
0.75	6
0.74	8
0.73	8
0.72	10
0.71	10
0.70	12
0.69	14
0.68	16
0.67	18
0.66	21
0.65	22
0.64	24
0.63	25
0.62	27
0.61	32
0.60	41
0.59	49
0.58	54
0.57	62
0.56	71
0.55	80
0.54	93
0.53	105
0.52	114
0.51	134
0.50	164
0.49	182
0.48	207
0.47	230
0.46	263
0.45	295
0.44	321
0.43	362
0.42	397
0.41	442
0.40	486
0.39	541
0.38	605
0.37	670
0.36	723
0.35	779
0.34	856

0.33	940
0.32	1017
0.31	1114
0.30	1187
0.29	1289
0.28	1403
0.27	1493
0.26	1587
0.25	1705
0.24	1826
0.23	1944
0.22	2062
0.21	2178
0.20	2311
0.19	2452
0.18	2556
0.17	2717
0.16	2871
0.15	3030
0.14	3177
0.13	3318
0.12	3480
0.11	3643
0.10	3780
0.09	3912
0.08	4037
0.07	4165
0.06	4314
0.05	4449
0.04	4573
0.03	4690
0.02	4802
0.01	4917
0.00	5026

Table A: Correlation cutoff and membership counts

Correlation cutoffs and associated membership counts. These are gene symbols where all of their correlational values (protein vs mRNA, mRNA vs CNV, and protein vs CNV) are above the given cutoff.

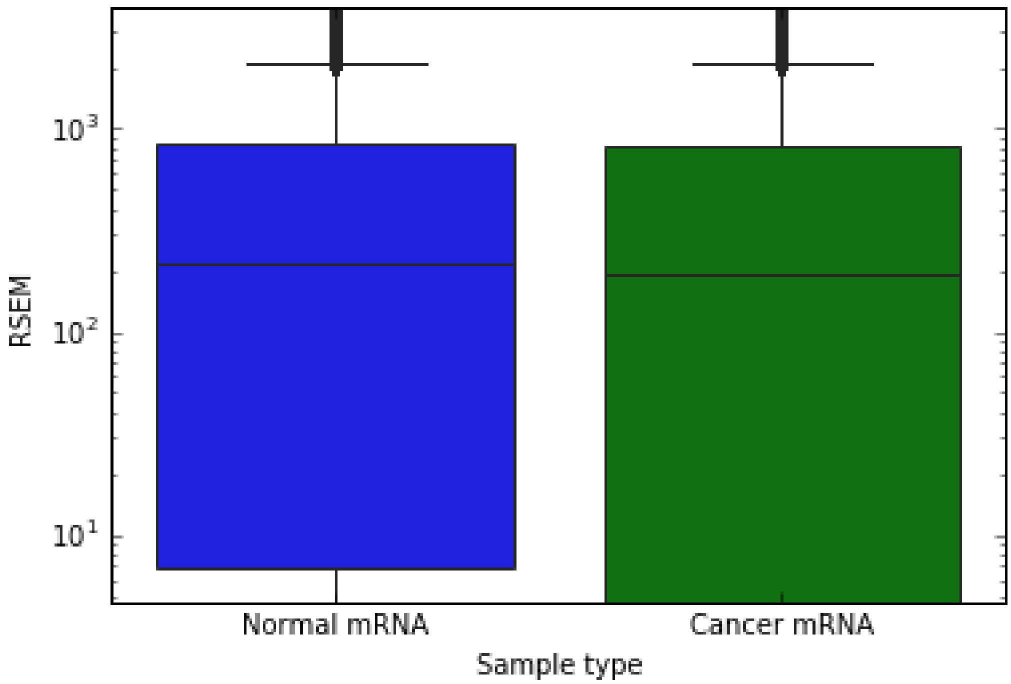

Fig B. Tumor-matched normal and tumor sample expression distributions

mRNA expression distributions for tumor-matched normal and tumor samples. Distributions are similar suggesting no batch effect or temporal confounder. This does not illustrate changes of a singluar genes expression between the two states, rather summary information of expression.

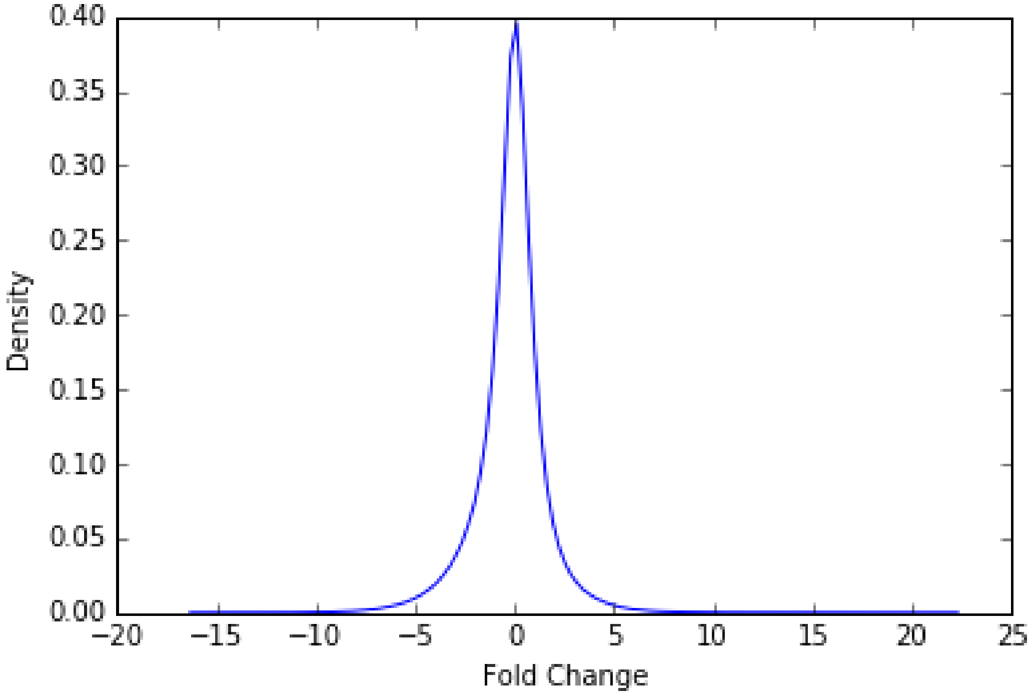

Fig C. mRNA log_2 fold change from normal median to cancer

mRNA log_2 fold change distributions illustrate classes of genes within patients that have high degrees of alteration. In this instance we can now identify genes with large log2 fold change differences from cancer to healthy states.

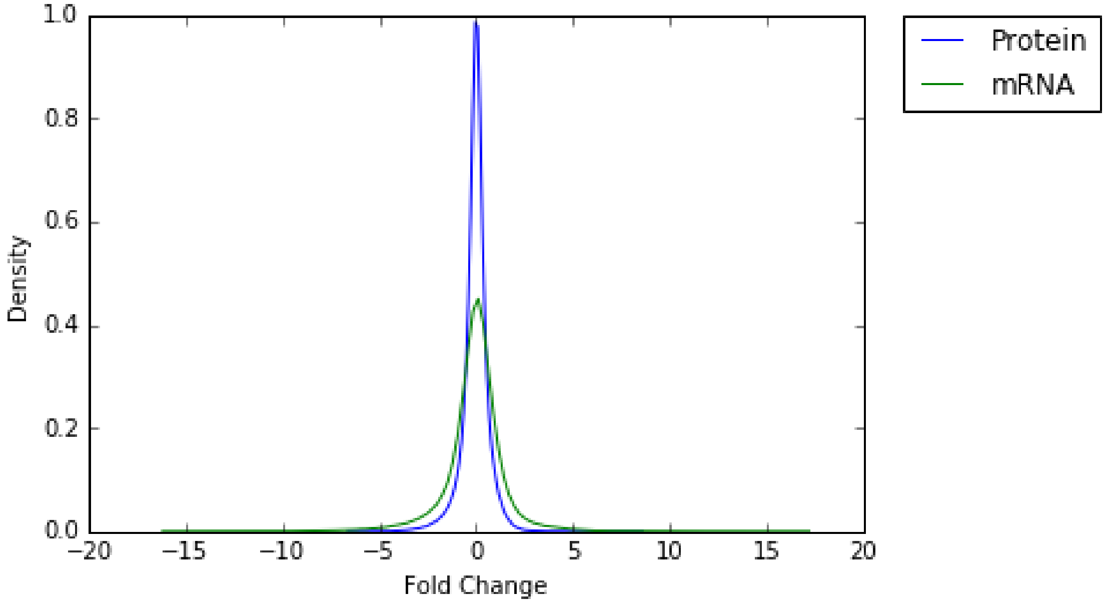

Fig D. Patient matched mRNA and protein fold change distributions

mRNA and protein fold change distributions illustrating similarity in a similar distribution with a mean around 0, however tails and quantiles are different.

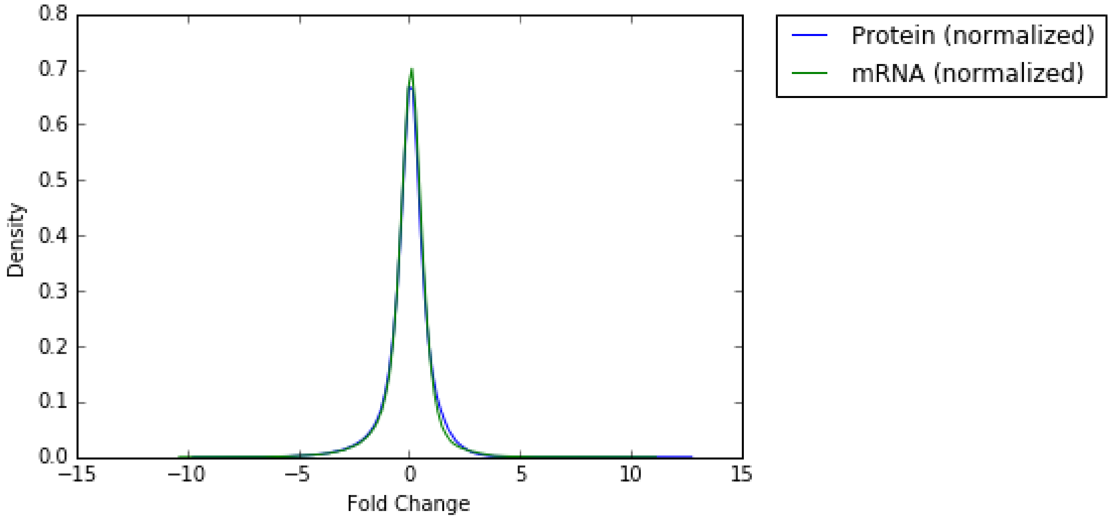

Fig E. Patient matched mRNA and protein fold change distributions after normalization

mRNA and protein fold change distributions after z-score normalization show same distributions.

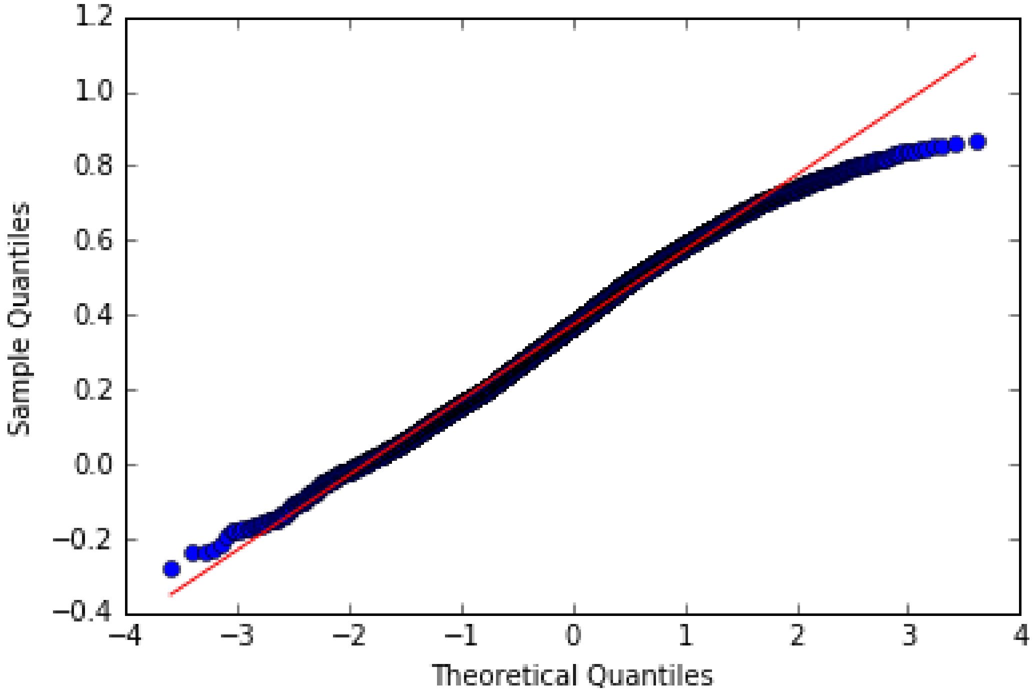

Fig F. QQ plot of mRNA vs protein fold change correlations

QQ plot illustrating tailed nature of the distribution of correlational scores. A D'Agostino's K-squared test rejected the null hypothesis that the data was Gaussian ($p < 0.005$). This indicates the data is non-normal, with a marginally lighter left tail and heavier right tail.

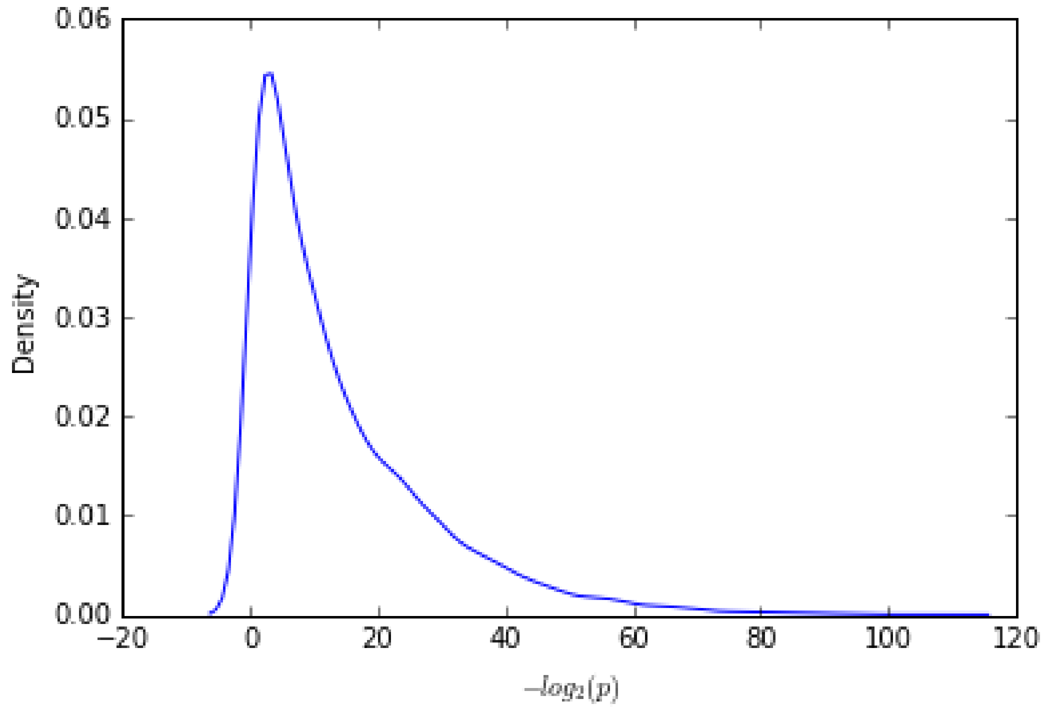

Fig G. Spearman correlation p value distribution between mRNA and Protein fold changes

Spearman correlation $-log_2$ p values were calculated between samples' mRNA and protein fold-change values.

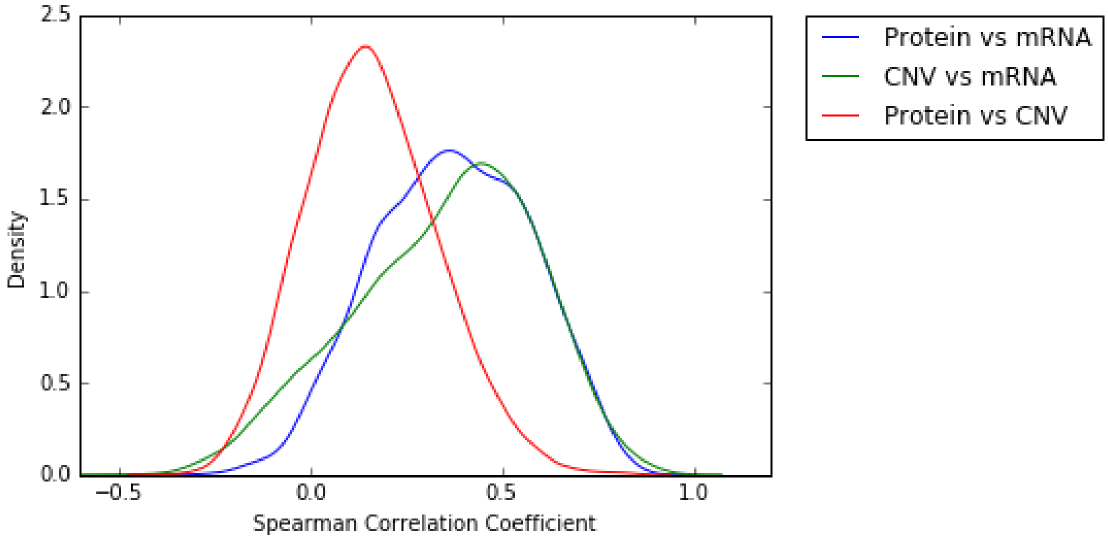

Fig H. Correlation coefficient distribution

Overall correlation coefficient distributions between genomic features emphasizes the decoupling of mRNA and CNV from overall protein production in the TCGA breast cancer samples.
